# *Stvb-i*, a Rice Gene Conferring Durable Resistance to *Rice stripe viru*s, Protects Plant Growth From Heat Stress

**DOI:** 10.3389/fpls.2020.00519

**Published:** 2020-05-08

**Authors:** Yuriko Hayano-Saito, Keiko Hayashi

**Affiliations:** NARO Central Region Agricultural Research Center, Tsukuba, Japan

**Keywords:** *Oryza sativa*, virus resistance gene, *Stvb-i*, *Rice stripe virus*, durable resistance, heat stress, plant growth, developmental homeostasis

## Abstract

Disease resistance is affected by temperature. A rice gene, *Stvb-i*, is known to have conferred sustained resistance to *Rice stripe virus* (RSV) despite global warming. *Stvb-i* protects plants from growth stunting caused by RSV. The underlying resistance mechanism is unclear. Here, *Stvb-i* showed stable RSV resistance for 20 years in laboratory experiments. This gene encodes a protein distinct from well-studied plant disease-resistance proteins. It has a domain homologous to the histidine kinase/heat-shock protein 90-like ATPase superfamily. Rice has three paralogous genes including *Stvb-i*. The genes are expressed mainly in meristematic tissues. In the initial period after viral inoculation, RSV multiplication enhanced *Stvb-i*, whereas *Stvb-i* suppressed RSV multiplication. *Stvb-i* silencing inhibited plant growth regardless of viral infection, and silencing of the other paralogous gene that located closely to *Stvb-i* caused morphological abnormalities. The results suggested that the *Stvb-i* and its paralogs are related to plant development; especially, *Stvb-i* supports meristem growth, resulting in plant growth stabilizing. Growth stunting in the *Stvb-i*–silenced plants was more severe under repetitive heat stress, suggesting that *Stvb-i* contributed to the attenuation of heat damage in plant development. The symptoms of RSV infection (chlorosis, wilting, stunting, fewer tillers, and defective panicles) were similar to those of heat damage, suggesting that RSV multiplication induces heat-like stress in meristematic cells. Our findings suggest that the mechanism of meristem growth protection conferred by *Stvb-i* allows plants to withstand both heat stress and RSV multiplication. The suppression of RSV multiplication by the *Stvb-i* function in meristems results in durable resistance.

## Introduction

Plants need to withstand abiotic and biotic stresses such as temperature fluctuations, water scarcity (drought), wind, excessive or insufficient light, and pathogens. Viral multiplication in plant meristems directly threatens plant survival because meristems are indispensable for plant growth and development. Plants have evolved sophisticated systems that allow them to avoid or reduce damage to meristems.

Many rice viruses inhibit plant growth (Ling, 1972; Hibino, 1996; Satoh et al., 2010, 2011, 2013; Budot et al., 2014). In particular, *Rice stripe virus* (RSV), an RNA virus causative of rice stripe disease, invades plants when they are sucked by the small brown planthopper (SBPH, *Laodelphax striatellus* Fallén) and greatly damages rice production, mainly in temperate East Asia, the world leading rice production area (Hibino, 1996; Otuka, 2013). In RSV-infected plants, typical leaf symptoms are discontinuous pale-yellow stripes, chlorotic streaks, and wilting (Ling, 1972). Systemic symptoms include stunted plant growth, fewer tillers, and defects in panicle formation and grain filling (Ling, 1972; IRRI Rice Knowledge Bank^1^). Infection at the seedling stage causes severe leaf and systemic symptoms, resulting in plant death (Ling, 1972; Hibino, 1996). Serious damage is supposed to be determined by where RSV multiplies and how it spreads. After invasion, RSV immediately migrates to meristematic tissue at the base of the seedling, which provides cells for the basic structure of the plant body, multiplies there and spreads systemically with active cell division (Sonku and Sakurai, 1973). RSV particles were observed in cells of the meristems including apical domes and leaf primordia (Takahashi et al., 2008).

An RSV resistance gene, *Stvb-i*, has been introgressed into *Oryza sativa* spp. *japonica* cultivars from the *indica* cultivar Modan (Hayano-Saito et al., 1998, 2000). The *Stvb-i*–harboring rice seedlings show milder symptoms than susceptible rice: faint discontinuous leaf stripes, reduced systemic symptoms, and decreased death by wilting (Washio et al., 1967, 1968). *Stvb-i* does not completely suppress RSV multiplication, and RSV is detectable at a low level in seedling base tissues including the meristem (Hayano-Saito, 2014).

Abiotic stresses influence plant resistance to viruses; some resistance genes are temperature sensitive (Wang et al., 2009; Kobayashi et al., 2014). A major type of plant resistance genes encodes nucleotide-binding site and leucine-rich-repeat (NBS-LRR) domain proteins, which elicit a hypersensitive response (Wang et al., 2009; Kobayashi et al., 2014). The genes for NBS-LRR proteins lose their function above a certain temperature (Samuel, 1931; Wang et al., 2009; Kobayashi et al., 2014). RSV multiplication is easily enhanced by short high-temperature treatment (42°C for 30 min) in *Nicotiana benthamiana* (Jiang et al., 2014). However, the hypersensitive response is not observed in *Stvb-i*–harboring plants (Hayano-Saito et al., 2000), and the effect of temperature has not been investigated.

Viral resistance is often rapidly weakened or overcome (Kobayashi et al., 2014). Interestingly, *Stvb-i*–harboring rice varieties have shown stable resistance for over 50 years in the paddy fields of Japan (Hayano-Saito, 2014). *Stvb-i* is located in the multi-allelic *Stvb* locus (Washio et al., 1968), and several resistant alleles have been reported (Hayano-Saito et al., 2000; Maeda et al., 2006; Wang et al., 2011; Wu et al., 2011; Zhang et al., 2011; Kwon et al., 2012). Among the allelic genes, *STV11* derived from an *indica* rice Kasalath has been isolated (Wang et al., 2014) but the relationships among the allelic genes remain unclear. To elucidate the durability of RSV resistance mediated by *Stvb-i*, we investigated the role of this gene in rice and its effects on RSV multiplication. We revealed the structure of the *Stvb-i* gene and found that it functions in developmental homeostasis, especially in meristematic tissues, contributing to the recovery from heat stress. Heat stress alleviation mediated by *Stvb-i* reduces RSV multiplication as well as leaf and systemic symptoms. Protection of meristems from stresses confers durable RSV resistance to S*tvb-i*–harboring plants. Our study helps to understand the intricate relationships between a plant and its pathogenic virus and provides important information on stress tolerance in meristems.

## Materials and Methods

### Plant Materials

Rice (*O. sativa*) cultivars Tsukinohikari, Asanohikari, and Musashikogane are RSV-resistant descendants of a resistant cultivar St No. 1 into which the RSV resistance gene *Stvb-i* of the *indica* rice cultivar Modan was introgressed; all three cultivars have the *Stvb-i* gene (Hayano-Saito et al., 1998; Hayano-Saito, 2002). Two RSV-susceptible *japonica* paddy cultivars, Nipponbare and Koganebare, were used as control cultivars in inoculation tests. Nipponbare is the standard rice cultivar used in the International Rice Genome Sequencing project. Koganebare is a progeny of Nipponbare; their properties are very similar, including stripe disease susceptibility (Koumura et al., 1980). Koshihikari, Kirara397, and Yuukara are also RSV-susceptible *japonica* paddy cultivars. Recombinant inbred lines (3,629 lines) at the F_7_ generation developed from a cross between Tsukinohikari and Koganebare were used to delimit the *Stvb-i* region; among them, RIL3245 was resistant and RIL484 was susceptible (Hayano-Saito, 2002). Yuukara and RIL484 were used in the complementation test. St No. 1, Tsukinohikari and Yuukara were used in the RNAi-mediated suppression test.

### Inoculation With RSV and Assessment of Resistance

To evaluate RSV resistance, bioassays using viruliferous SBPHs in the seedling test (Washio et al., 1967, 1968) were conducted as described elsewhere (Hayano-Saito et al., 1998). The susceptible cultivars were Nipponbare, Koganebare, Koshihikari, Kirara397, and Yuukara; resistant cultivars were St No. 1, Tsukinohikari, Asanohikari, and Musashikogane. A colony of the vector (infective ability >60%) derived from viruliferous SBPHs collected in the Kanto district (Japan) was obtained from the former National Agriculture Research Center (currently called the Central Region Agriculture Research Center, Tsukuba, Japan) in 1990. To maintain the high infective ability of the colony, RSV-infective SBPHs were reselected two or three times a year by inoculation tests or a serological method (Washio et al., 1968). The SBPH population used in the bioassays recirculated the virus between rice and the insects. RSV-inoculated plants were grown in a glasshouse and plants with symptoms were counted 4–5 weeks after inoculation. Bioassays were carried out in 1990–1992 at Tsukuba (National Institute of Agrobiological Science, 36°03′10.6″N, 140°10′15.2″E) and 1996–2009 at Sapporo (Hokkaido Agricultural Research Center, 43°00′92.8″N, 141°41′15.0″E) in Japan. Weather data for Tsukuba were obtained from the weather data acquisition system of the Institute for Agro-Environmental Sciences^2^ and the data for Sapporo were obtained from the meteorological observation system at the National Agricultural Research Center for Hokkaido Region since 1990^3^.

The ratio of diseased plants represents the ratio of the percentage of diseased plants in the tested population to that in the population of Nipponbare (control). Each plant with chlorosis (Supplementary Figure S1A) was counted as diseased. Reactions to RSV were evaluated by the “ratio of disease-rating index” (RDRI) as described elsewhere (Washio et al., 1967, 1968) (Supplementary Figure S1B). Based on the RDRI, the cultivars and lines were classified as resistant (R, RDRI ≤ 30), moderately resistant (M, 30 < RDRI ≤ 60), or susceptible (S, RDRI > 60). The RDRI data were obtained from six replications of the complementation test and from three replications of the RNAi-mediated suppression test. Thirty plants were used in one replication.

*Rice stripe virus* inoculation for tissue staining and RNA analysis was performed as follows: rice seedlings at the 1.0–1.5-leaf stage were exposed to 10 viruliferous SBPHs per plant at 25–27°C in a growth chamber, and the insects were removed after 24 h. Non-viruliferous SBPHs were used for mock-inoculation of control plants. Plants were kept in the growth chamber until RNA preparation.

### Immunohistochemistry

Paraffin sections were prepared from 3–5 mm pieces of the bases of rice seedlings. Seedling bases at 5–10 days after RSV inoculation were sampled. Sections were stained with a Vectastain ABC-AP kit (Vector Laboratories^4^) and antibody against RSV (rabbit IgG, Japan Plant Protection Association), and were observed under a digital microscope VHX-5000 (Keyence^5^) using without imaging modification.

### Sequencing, Gene Annotation, and Sequence Similarity Search

An ABI310 genetic analyzer and a BigDye Terminator Cycle Sequencing Kit version 3.1 (Applied Biosystems^6^) were used for DNA sequencing. The *Stvb-i* genomic region sequences of St No. 1 and Nipponbare provided by the Rice Genome Project (National Institute of Agrobiological Sciences, Tsukuba, Japan) were used for marker development and sequence comparison. Candidate *Stvb-i* ORFs were predicted and annotated with the Rice Genome Automated Annotation System (Sakata et al., 2002). Protein domain analysis of Stvbi was performed with InterPro^7^. Paralogous gene information was obtained from the MSU Rice Genome Annotation Project^8^ (Kawahara et al., 2013). The sequences of Stvbi paralogs were aligned by using Genetyx-Mac (ver. 18, Genetyx^9^).

### Genotyping Analysis

Cleaved amplified polymorphic sequence, simple sequence repeat, and simple sequence length polymorphism markers were newly developed from the comparison between the genomic sequences of St No. 1 and Nipponbare (Supplementary Table S1). These PCR-based markers and restriction fragment length polymorphic markers (Hayano-Saito, 2002) were used for fine genotyping analyses of recombinant inbred lines. DNA extraction and genotyping analyses were performed as described elsewhere (Hayano-Saito et al., 1998; Saito et al., 2010).

### DNA Constructs and Rice Transformation

The DNA of the *Stvb-i* region covered by the BAC clone St0014J19 (Supplementary Figure S2) was purified using a Large-Construct Kit (Qiagen^10^). Genomic DNA of St No. 1 was purified as described elsewhere (Hayano-Saito et al., 1998), partially digested with *Sau*3AI, and size-fractionated in the 20–25-kb range by ultra-centrifugation (5–25% stepwise gradient of NaCl containing 3 mM EDTA, 200,000 × *g*, 4.5 h at 20°C, Hitachi RPS40T rotor). A transformation-competent artificial chromosome (TAC) library was constructed in *Bam*HI-digested pYLTAC7 as described elsewhere (Liu et al., 1999). Clones carrying candidate ORFs were selected from the library and introduced into ElectroMAX *Agrobacterium tumefaciens* LBA4404 (Invitrogen^11^). Plants were transformed with *A. tumefaciens* as described elsewhere (Ozawa, 2009). Transgenic lines with the StD fragment (accession no. LC157868) evaluated for RSV resistance in the complementation test were derived from RIL484 (DR201, DR202, and DR204), and Yuukara (DY401, DY402, DY505, DY508, and DY510).

For RNAi-mediated suppression of *ST07i* (the candidate for *Stvb-i*), an internal fragment, NK5 trigger (370 bp, Supplementary Figure S3), was amplified with the NK5 primer set (Supplementary Table S1) and was used as an inverted repeat to transcribe the trigger dsRNA. The fragment was inserted into the pANDA vector as described elsewhere (Miki and Shimamoto, 2004). The RNAi construct was introduced into St No. 1, Tsukinohikari, and Yuukara as described above. The NK5-RNAi–silenced lines St507 and St509 were derived from St No. 1; Tu518 and Tu520 from Tsukinohikari; and Yu5001, Yu5002, and Yu5008 from Yuukara. Four transgenic lines (St507, St509, Tu518, and Tu520) were used for RNAi-mediated suppression tests; St507, St509, Yu5001, Yu5002, and Yu5008 were used for gene expression analyses and plant phenotyping. For stable stress tolerance evaluation, we used T_6_ generation transgenic lines with fixed plant properties, instead of unstable T_2_ generation transgenic lines.

### RNA Preparation

**FIGURE 1:**
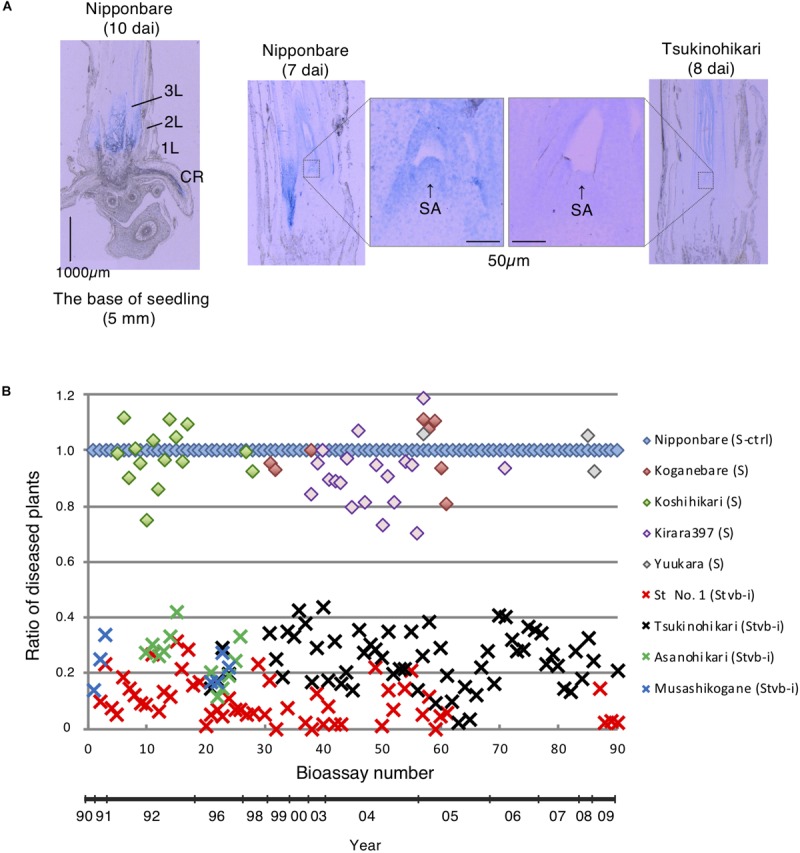
**(A)** Viral localization in meristematic issue by immunostaining. 1L, 1st leaf; 2L, 2nd leaf; 3L, 3rd leaf; CR, crown root; SA, shoot apex. **(B)** Bioassays for rice stripe virus resistance conferred by *Stvb-i* from 1990 to 2009. Reaction to the virus is presented as the ratio of diseased plants. The ratio of diseased plants represents the ratio of the percentage of diseased plants in the tested population to that in the population of Nipponbare. In each bioassay, 30–180 plants per cultivar were tested. Nipponbare was used as a susceptible control cultivar (S-ctrl).

Total RNA was extracted by using TRIZOL Reagent or TRIZOL Plus RNA Purification Kit (Invitrogen) from 3–5-mm pieces of the bases of seedlings (including leaf and root primordia and shoot apices, Figure 1A) and tillers (including leaf primordia and shoot apices), 5-mm pieces of the 3rd leaf blades of seedlings, 10-mm pieces of flag leaf blades, 10-mm-long young whole panicles (including multiple primordia in each glumous flower) in the sheath, and 100 mg of callus prepared for transformation as above. Etiolated seedlings were grown at 25–27°C in a plant growth chamber in the dark. Seedling bases were sampled from six plants and other parts from three plants.

### Cloning of *Stvb-i* and Its Paralogs

For *Stvb-i* cloning, total RNA was extracted from 10 seedlings. The 5′ end of the transcript was identified using 5′-rapid amplification of cDNA ends (RACE) Core Set (Takara-Bio^12^). Full-length cDNA was generated using an RNA LA PCR Kit (Takara-Bio), cloned using a Mighty TA-Cloning Kit (Takara-Bio), and sequenced. The transcripts of *Stvb-i* paralogous genes were identified on the basis of database information, amplified from suitable tissues as above and sequenced.

### Expression Analyses of Viral and Rice Genes

All primers and the sizes of the amplified fragments are listed in Supplementary Table S2. To analyze the expression of rice and viral genes, reverse transcription–polymerase chain reaction (RT-PCR) was carried out using an RNA-PCR Kit (Takara-Bio). The RSV genome encodes the following proteins: RdRp, RNA-dependent RNA polymerase; P2, membrane-associated protein; Pc2, membrane glycoprotein; NS3, non-structural protein 3; CP, coat protein; P4, major non-structural protein; and Pc4, non-structural protein 4 (Zhu et al., 1991, 1992; Takahashi et al., 1993; Toriyama et al., 1994). The primers for RSV genes were developed using the following sequences: accession nos. D31879.1 (*RdRp* on RNA1), D13176.1 (*P2* and *Pc2* on RNA2), D01094.1 (*NS3* and *CP* on RNA3), and D01039.1 (*P4* and *Pc4* on RNA4). Rice glyceraldehyde 3-phosphate dehydrogenase (*OsGAPDH*, accession no. AK064960) was used as an internal reference gene.

To measure *Stvb-i* expression in RSV-infected plants, seedlings were sampled every day at noon. Total RNA was prepared from six seedlings inoculated with or without RSV. Quantitative RT-PCR was conducted on an ABI7500 Real-time PCR System (Applied Biosystems) using a SYBR PrimeScript RT-PCR Kit (Takara-Bio). *Stvb-i* expression levels were determined by the standard curve method; rice *OsGAPDH* (see above) was used as an internal reference gene. To compare gene expression in heated and non-heated rice plants, total RNA was prepared from four seedlings per treatment. Quantitative RT-PCR was conducted on a Roche Lightcycler 480 Real-Time PCR system (Nippon Genetics^13^) using Thunderbird SYBR qPCR Mix (Toyobo^14^). The results were analyzed by the ΔΔCT method; rice *OsGAPDH* was used as an internal reference gene. The mean relative expression levels and the SEM (*n* = 3) were calculated using the statistical package BellCurve for Excel (Social Survey Research Information^15^). A Tukey–Kramer multiple comparison test was used for data analysis.

### Plant Height and Tiller Number

Two *Stvb-i*–silenced lines (St507 and St509) and three *Stvb-i*–complemented lines (Yu5001, Yu5002, and Yu5008) were used for plant phenotyping as follows.

To investigate the relationship between plant growth and RSV inoculation, St No. 1 and *Stvb-i*–silenced lines (15 plants per line; 15 plants per dish of 13 cm in diameter and 3 cm in depth) were used. Seedlings were inoculated with RSV at 1.5-leaf stage (6 days after germination) and the aboveground height was measured at 36 days after germination. Susceptible Nipponbare was used as reference.

For tiller counting, *Stvb-i*–silenced and -complemented lines of St No. 1 and Yuukara (15 plants per line; 3 plants per 15-cm diameter pot) were used. Non-infected plants were grown under normal growth conditions from May to August in Tsukuba, Japan. The tillers were counted 54 days after germination (vegetative stage) and 84 days after germination (heading stage).

To investigate the role of *Stvb-i* in heat stress, *Stvb-i*–silenced lines and St No. 1 were used. The aboveground height of 20 RSV-uninfected plants was measured from day 0 (start) to day 10. Leaf stage was measured from day 0 to day 11 with reference to Hoshikawa (1989).

All data were analyzed by a one-way or two-way analysis of variance (ANOVA), and if necessary a Tukey–Kramer multiple comparison test.

### Heat Treatment of Rice Seedlings

Healthy seeds with coats removed were surface-sterilized with sodium hypochlorite solution (2.5% effective chlorine concentration), washed several times with sterile distilled water, and soaked in sterile distilled water at 20°C for germination. Germinated seeds were planted in soil and seedlings were grown at 25°C (16 h, light) and 20°C (8 h, dark) in a controlled plant growth chamber. Rice seedling growth declines above 35°C (Krishnan et al., 2011). For observation of plant appearance during the whole growth period, *Stvb-i*–silenced and *-*complemented plants were exposed at 35–37°C for 4 days at 1st-leaf stage (4 days after germination) and were grown in a plant growth chamber for 7 days and then in a glasshouse. Similar observations were performed three times. To investigate the role of *Stvb-i* in heat stress, 20 seedlings of each of the silenced lines and WT St No. 1 at the 1st-leaf stage were left untreated or were heat-treated at 38°C for 2 days once or three times, and then grown at 25°C (16 h, light) and 20°C (8 h, dark) in a controlled plant growth chamber. The plant height and leaf stage were determined (see section “Plant Height and Tiller Number”).

For expression analysis, *Stvb-i*–silenced lines and St No. 1 were used. Germinated seeds were placed on 0.4% gellan gum containing 1/5 Murashige–Skoog medium in a plastic box for plant tissue culture. The seedlings were heat treated at 38°C for 12 h at the 1.5-leaf stage. Meristematic tissues were sampled at 0, 6, 12, 18, 24, 36, 48, and 60 h from the start of heat treatment (at 09:00 h), and RNA preparation and analysis were performed as described above.

## Results

### RSV Localization and Durability of *Stvb-i*–Mediated Resistance

In an RSV-resistant cultivar Tsukinohikari at 10 days after RSV inoculation, viral concentration at the seedling base is low and viral spread to the upper leaves is suppressed as compared to a susceptible cultivar Nipponbare (Hayano-Saito, 2014). We used an immunohistochemical technique to examine the localization of RSV in the base tissue of rice plants (Figure 1A). In Nipponbare, RSV spread mainly in the upper part of the 1st node at 10 days after RSV inoculation; the signals were especially dense in the primordium and shoot apex at 7 days after RSV inoculation. This indicates that RSV is present and propagates in meristems, and then spreads to other plant tissues and organs. In Tsukinohikari harboring *Stvb-i*, the signals were strongest in the upper part of the developing leaves in the sheath, were weaker in the base part of the leaves, and were very weak in the meristem. These data suggest that, although RSV multiplies in young leaves, the increase in the number of virus particles is suppressed in the meristem of Tsukinohikari, i.e., *Stvb-i*–mediated resistance suppresses RSV propagation in meristematic tissue.

We evaluated the severity of RSV infection on the basis of leaf symptoms, mainly chlorosis (yellowing) and twisting (Supplementary Figure S1A), in four resistant cultivars (St No. 1, Tsukinohikari, Asanohikari, and Musashikogane) and five susceptible cultivars (Nipponbare, Koganebare, Koshihikari, Kirara397, and Yuukara) in 90 bioassays performed over 20 years (Figure 1B). In bioassays, we used the SBPH population recirculated the virus between rice and the insects.

The ratio of diseased plants was considerably lower in the presence of *Stvb-i*; in all resistant rice cultivars, it rarely exceeded 0.4 (Figure 1B). The resistance of St No. 1 was slightly stronger than those of its progeny, Tsukinohikari, Asanohikari, and Musashikogane. The pathogenicity of RSV did not change over the 20 years. These observations indicate that *Stvb-i*–mediated resistance is durable.

### Isolation of the *Stvb-i* Gene

**FIGURE 2:**
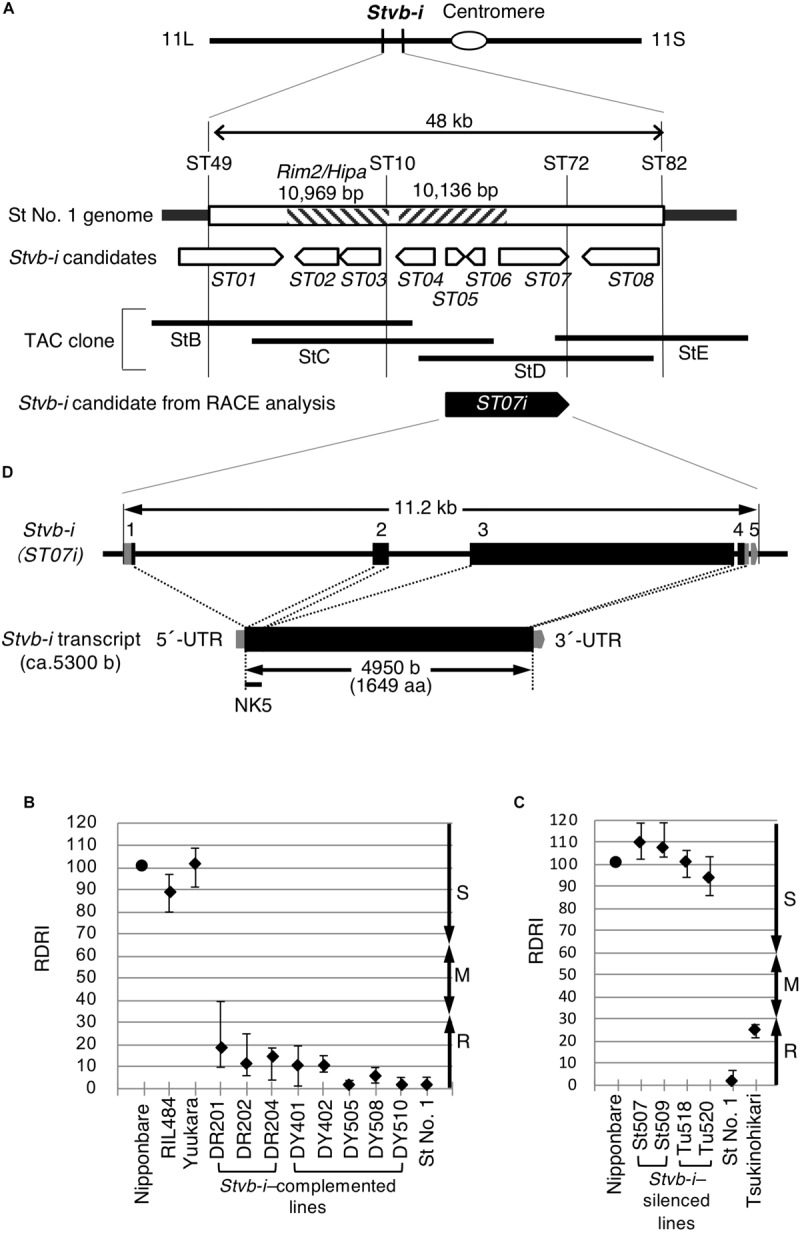
Map-based cloning of the *Stvb-i* gene. **(A)** Fine physical map of the 48-kb *Stvb-i* region, structure of the *Stvb-i* locus, and the *Stvb-i* candidate genes. Pentagons represent the predicted candidate genes and the identified candidate gene, *ST07i*, and their direction. A *Rim2/Hipa* transposon is present in St No. 1 but not in Nipponbare. In the Nipponbare genome, a 10,136-bp segment is substituted by a 6,383-bp segment with low sequence identity. **(B)** Average disease rating indexes of StD lines relative to that of Nipponbare (RDRI) (*n* = 6; capped bars show the ranges). Control cultivars were Nipponbare (susceptible) and St No. 1 (resistant). **(C)** Average RDRIs of RNAi lines (*n* = 3) compared with Nipponbare and St No. 1. **(D)** Structure of *Stvb-i*. Coding regions are shown in black and the untranscribed regions (UTRs) in grays. NK5 represents a trigger fragment for the RNAi construct.

We isolated *Stvb-i* by map-based cloning. Genetic analysis using recombinant inbred lines delimited the *Stvb-i* locus to a 48-kb interval between markers ST49 and ST82 on the long arm of chromosome 11 (Figure 2A and Supplementary Figure S2). We compared this region with the genome of the susceptible cultivar Nipponbare. The *Stvb-i*–harboring RSV-resistant cultivar St No. 1 had a *Rim2/Hipa* transposon (Wang et al., 2003). A 10,136-bp segment was substituted by a 6,383-bp segment, with low sequence identity between the two segments. Mapping identified eight putative genes (*ST01*–*ST08*) as candidates for *Stvb-i* (Figure 2A). Each candidate gene region alone failed to confer RSV resistance, however, introduction of the StD fragment (accession no. LC157868; Figure 2A) conferred resistance in susceptible RIL484 and Yuukara (Figure 2B).

The StD fragment covered the coding regions of three predicted candidate genes, *ST05*, *ST06*, and *ST07.* Using RACE analyses, we found another candidate gene, *ST07i*, in the StD fragment (Figure 2A). Suppression of *ST07i* expression by RNA silencing made the transgenic lines of resistant cultivars susceptible to RSV (St507, St509 in the St No. 1 background; Tu518, Tu520 in the Tsukinohikari background; Figure 2C). St507 and St509 tended to be higher RDRI values than did Tu518 and Tu520 (Figure 2C). Thus, the results of bioassays (Figures 1B, 2C) suggest that the genetic background affects RSV resistance level.

Thus, we concluded that *ST07i* is *Stvb-i* (Figure 2D). This gene located at LOC_Os11g31480 spans 11.2 kb, has five exons and encodes a 1,649-aa protein that has no NBS-LRR domain but has a domain homologous to the histidine kinase/HSP90-like ATPase superfamily (IPR036890). The result indicates that *Stvb-i* differs from the RSV-resistance gene *STV11* (*OsSOT1*, LOC_Os11g30310), which is derived from an *indica* rice Kasalath and encodes an enzyme sulfotransferase (Wang et al., 2014).

### Viral RNA Production and *Stvb-i* Expression

**FIGURE 3:**
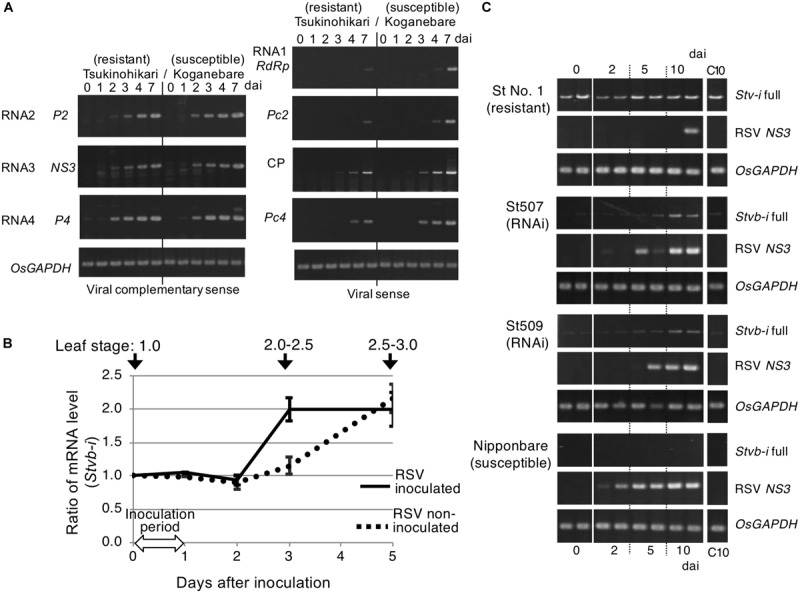
Expression of RSV genes and *Stvb-i* in seedling meristems. **(A)** Expression patterns of all seven genes present in the RSV genome (*RdRp*, *P2*, *Pc2*, *NS3*, *CP*, *P4*, and *Pc4*) in the cultivars Tsukinohikari (resistant) and Koganebare (susceptible). dai, days after inoculation. **(B)** Quantitative RT-PCR analysis of *Stvb-i* expression in Tsukinohikari. Transcript levels are shown relative to the level on day 0 (start of RSV inoculation), and are means ± SEM (*n* = 3). **(C)** Expression of *Stvb-i* and RSV *NS3* in the *Stvb-i*–silenced transgenic T_2_ lines St507 and St509. C10, non-inoculated plants of the same type at 10 dai were used as controls.

Three days after inoculation, propagation of RSV particles in seedling base tissues including the meristem is higher in susceptible rice than in resistant rice (Hayano-Saito, 2014). The abundance of viral RNA species encoded both in the viral sense (*RdRp*, *Pc2*, *CP*, and *Pc4*) and complementary sense (*P2*, *NS3*, and *P4*) increased more slowly in a resistant cultivar than in a susceptible cultivar (Figure 3A). The viral sense RNAs were detected clearly later (by 3 days for *RdRp* and *Pc2*; by 1 day for *CP* and *Pc4*) in the resistant cultivar than in the susceptible cultivar. Thus, although RSV is able to multiply in both resistant and susceptible rice cultivars, multiplication is slower in resistant rice. This result is consistent with the immunohistochemical observation (Figure 1A).

The delay of RSV RNA replication in the resistant cultivar Tsukinohikari occurred within a few days after viral inoculation. We examined *Stvb-i* expression in the initial 0–5 days after RSV inoculation in Tsukinohikari (Figure 3B). *Stvb-i* was expressed before RSV inoculation (at day 0) (Figure 3B). In non-inoculated seedlings, the level of *Stvb-i* mRNA gradually increased from day 2 to day 5 to twice the initial level. In contrast, it increased sharply on day 3 upon RSV inoculation and then plateaued; the level on day 5 was similar in non-inoculated and inoculated seedlings. *Stvb-i* upregulation on day 3 was too late to halt viral RNA replication because RSV had already started multiplying by this time (Figure 3A). However, it might be sufficient to suppress further RSV multiplication.

We next investigated *Stvb-i* expression in the silenced lines derived from St No. 1, in which the effect of *Stvb-i* was slightly stronger than in Tsukinohikari (Figures 1B, 2C). In the *Stvb-i*–silenced lines St507 and St509, *Stvb-i* expression was almost undetectable before RSV inoculation and for up to 5 days after inoculation, but increased slightly 5–10 days after RSV inoculation (Figure 3C), probably because of the presence of the viral RNA-silencing suppressor NS3 (Xiong et al., 2009). In spite of restored *Stvb-i* expression, the infected plants were severely damaged (Figure 2C). This result indicates that resistance is not affected by restoration of *Stvb-i* expression after 5 days post-inoculation.

Taken together, the results suggest that Stvbi presence in the seedling meristem prior to RSV invasion is required for RSV resistance and that *Stvb-i* is upregulated in response to RSV multiplication.

### Genes Paralogous to *Stvb-i*

**FIGURE 4:**
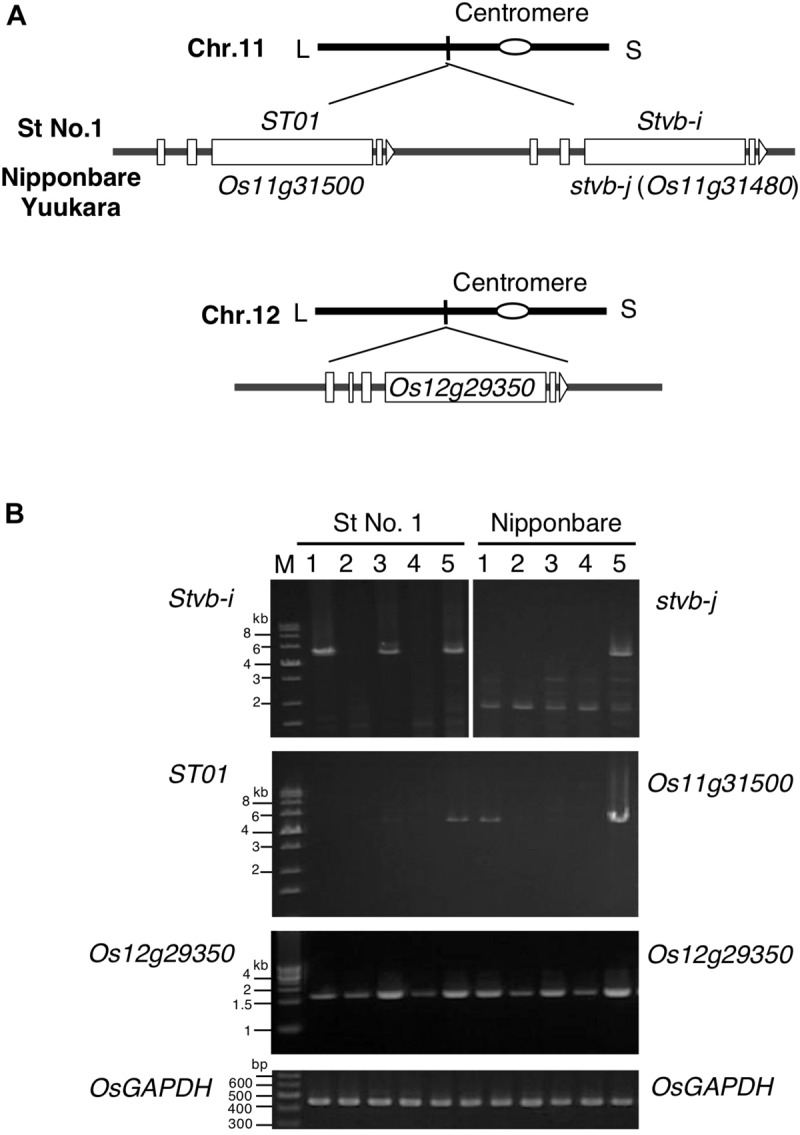
*Stvb-i* paralogous genes and their expression. **(A)** The structures of the *Stvb-i* paralogs *ST01*, *stvb-j* (*Os11g31480*), *Os11g31500*, and *Os12g29350*. These four genes are shown according to sequence information in the database of the MSU Rice Genome Annotation Project (http://rice.plantbiology.msu.edu). L, long arm; S, short arm. **(B)** Expression of *Stvb-i* paralogs. Lanes 1, seedling bases; 2, mature 3rd-leaf blades; 3, tiller bases; 4, mature flag-leaf blades; 5, young panicle (10 mm in length); M, molecular DNA size marker: Gene Ladder 100 (0.1–2 kbp, Nippongene) for *OsGAPDH* and Perfect DNA Markers (0.5–12 kbp, Novagen) for the others.

While comparing the sequences of the *Stvb-i* region on rice chromosome 11 between St No. 1 and Nipponbare, we found two paralogous genes in each cultivar: *ST01* and *Stvb-i* in St No. 1, and *ST01*-allelic *Os11g31500* and *Stvb-i*–allelic *stvb-j* (*Os11g31480*) in Nipponbare (Figure 4A). The amino acid sequence similarity among the four deduced proteins exceeded 90%. A BLAST search identified an additional Stvbi paralog encoded by *Os12g29350* on chromosome 12 of Nipponbare (Figure 4A). All five paralogs had an N-terminal domain homologous to that in histidine kinase/Hsp90-like ATPase superfamily proteins, implying that these proteins have the same or similar functions.

The *Stvb-i*–paralogous genes showed different expression patterns in plant tissues (Figure 4B). In resistant St No. 1, *Stvb-i* was expressed in plant parts containing meristems (seedling base, axillary tiller base, and 10-mm-long young panicle), and was highly expressed in calluses, but not in elongated leaf blades (Figure 4B and Supplementary Figure S4). The *stvb-j* gene of susceptible Nipponbare was expressed only in young panicles. *ST01* of St No. 1 and *Os11g31500* of Nipponbare were expressed mainly in young panicles. *Os11g31500* was also expressed at a low level in the seedling base, but *ST01* was not. *Os12g29350* was expressed in all tested tissues of both St No. 1 and Nipponbare; the expression was stronger in immature plant parts containing meristems than in mature tissues such as leaf blades (Figure 4B). Only *Stvb-i* was expressed specifically in seedling and tiller meristems, where RSV multiplies.

### Role of *Stvb-i* and Paralogous Genes

#### Phenotypes of *Stvb-i*–Silenced Lines

**FIGURE 5:**
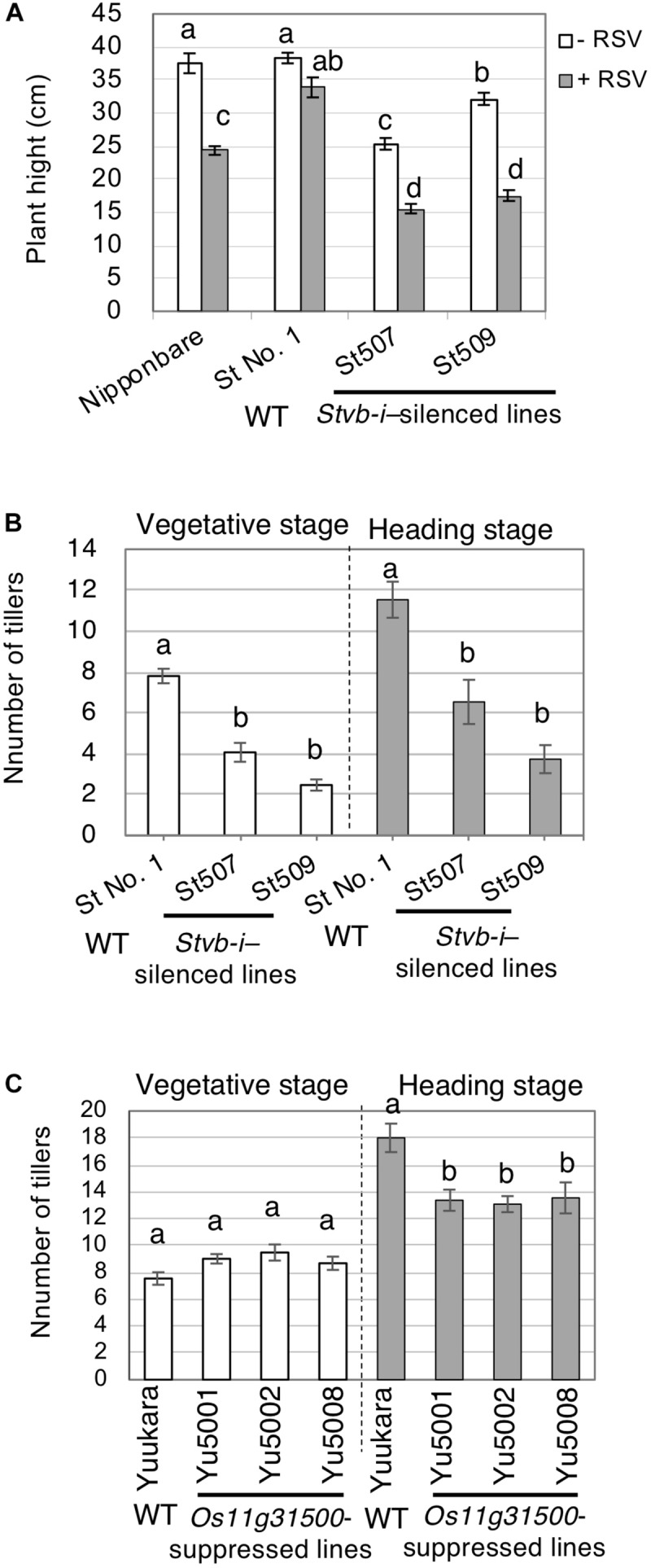
Role of *Stvb-i* and *Stvb-i* paralogous genes in plant growth. **(A)** Height of aboveground parts of rice plants at 36 days after planting. **(B)** Average number of tillers in non-inoculated plants of *Stvb-i*–silenced lines at the vegetative and heading stages. **(C)** The average number of tillers at the vegetative and heading stages in three non-inoculated RNAi lines (*Os11g31500*-suppressed lines), Yu5001, Yu5002, and Yu5008. In all three panels, different letters indicate significant difference (Tukey–Kramer test, *p* < 0.01, *n* = 15). WT, wild type.

*Rice stripe virus* infection caused more severe stunting in the RNAi lines than in WT St No. 1, and both lines were significantly stunted regardless of RSV (Figure 5A). There was an interaction between RSV infection and rice cultivar or line in plant height (*df* = 3, *MS* = 154.03, *F* = 9.720, *P* = 0.000 in two-way ANOVA). Non-inoculated mature plants of these lines also had fewer tillers (Figure 5B) and showed drying of mature (elongated) leaves and bending of the lamina joint (Supplementary Figure S5A). These results suggest that *Stvb-i* has an important role in rice development.

#### Phenotypes of *Os11g31500*-Suppressed Lines

We examined the phenotypes of *Os11g31500*-suppressed lines, Yu5001, Yu5002, and Yu5008. The expression of the paralogous gene *Os11g31500* was suppressed by RNAi generated using the NK5 construct (Supplementary Figures S3, S6), which suggests that *Os11g31500* is a functional analog of *Stvb-i* in the susceptible cultivar Yuukara. Although plant height was not significantly affected in the RNAi-suppression lines (data not shown), the tiller number was decreased significantly in all three lines at the heading stage, but not at the vegetative stage (Figure 5C). Long upper glumes were frequently observed in the *Os11g31500*-suppressed lines (Supplementary Figure S7). Together with the expression pattern of *Os11g31500* (Figure 4B), these results suggest that *Os11g31500* is primarily involved in rice development at the reproductive stage.

### Role of *Stvb-i* in Heat Stress

The *Stvb-i*–silenced plants seemed to be affected by temperature: their growth was suppressed more strongly in hot summers at Tsukuba in 2015 (36°03′N; maximum temperature, 30.2°C in July and 30.4°C in August) than in mild summers at Sapporo in 2009 (43°00′N; maximum temperature, 21.8°C in July and 24.1°C in August); leaf drying, chlorosis and lamina joint bending were observed at both locations, and panicle malformation was observed at Tsukuba in the *Stvb-i*–silenced line (Supplementary Figure S5). We hypothesized that the effect of *Stvb-i* silencing on plant growth is particularly strong under heat stress.

**FIGURE 6:**
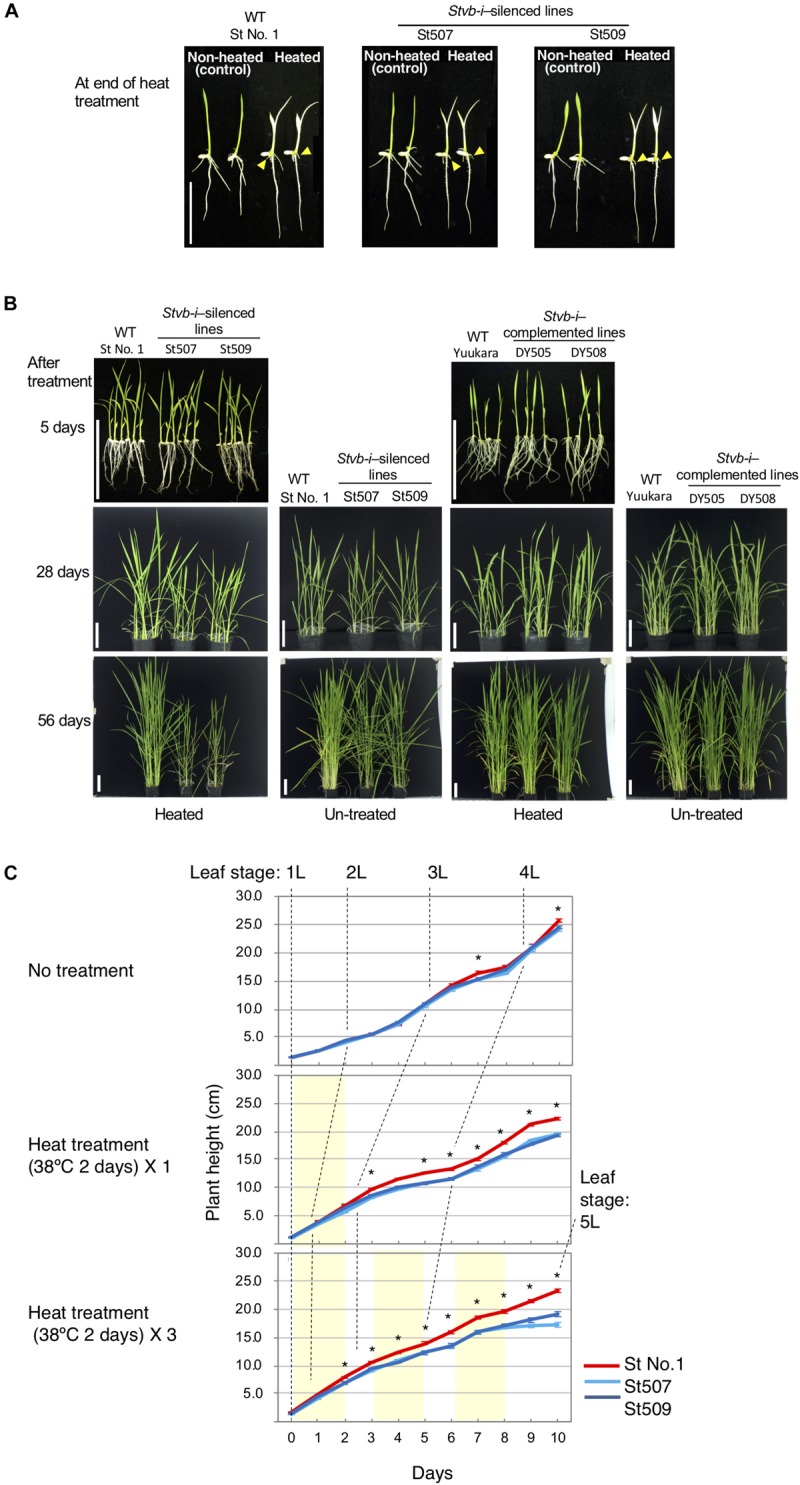
Plant phenotypes of rice plant exposed to high temperature. **(A)** Seedlings of *Stvb-i*–silenced lines and the WT St No. 1 at the end of heat treatment at 35–37°C for 4 days at the 1st-leaf stage. Heat-treated plants exhibited chlorosis and short crown roots (yellow arrowheads), whereas non-heated seedlings did not. Scale bar: 3 cm. **(B)** Appearance of *Stvb-i*–silenced plants and *Stvb-i*–complemented plants at 5, 28, and 56 days after the end of heat treatment (the same condition to panel **A**). Upper panel; representative heat-treated seedlings grown at 25°C in a growth chamber for 5 days after the end of the heat treatment. Middle and lower panels; plants grown for 28 and 56 days, respectively, after the heat treatment. Untreated plants were employed as controls for heated plants. Scale bars: 10 cm. **(C)** Plant height of *Stvb-i*–silenced lines (St507 and St509) and the WT St No. 1 exposed to high temperature. Seedlings of at the 1st-leaf stage were left untreated or were treated at 38°C for 2 days once or three times. Light yellow shading represents heating periods. Asterisks show significant differences between both *Stvb-i*–silenced lines and WT (Tukey–Kramer test, *p* < 0.01, *n* = 20).

Heat treatment (4 days at 35–37°C at the 1st-leaf stage) promptly caused chlorosis on whole seedling and short crown root (Figure 6A). Five days after the end of treatment, yellowing of leaves and slowed root development were alleviated, and the difference in plant phenotypes was unobservable between *Stvb-i*–silenced lines (St507 and St509) and the wild-type St No. 1 (Figure 6B). However, the silenced lines showed stunted growth up to 28 days after the end of the heat treatment in comparison with the wild type and non-heated plants, which recovered within an overall shorter period (Figure 6B). At 56 days after the heat treatment, the silenced lines were still stunted, whereas the treated and un-treated WT plants grew similar to each other (Figure 6B). The same heat stress experiment was performed on the *Stvb-i*–complemented lines (DY505 and DY508). In contrast to the *Stvb-i*–silenced lines, the complemented lines grew without stunting (Figure 6B). The silenced plants could not recover from heat damage suffered at the seedling stage, suggesting a positive effect of *Stvb-i* on growth of heated seedlings.

We exposed 1st-leaf-stage seedlings of the silenced lines to 38°C for 2 days once or three times and examined the height of the seedlings in detail. Heat treatment accelerated the leaf stage progress (Figure 6C), but there was no significant difference in leaf stage at day 11 between the silenced lines and the WT in each treatment (Supplementary Table S3). In the silenced lines, stunting appeared immediately after the heat treatment, whereas non-heated seedlings grew similarly with or without *Stvb-i* up to 3-leaf age (Figure 6C). The growth difference between the *Stvb-i*–silenced lines and the WT increased with each exposure to high temperature (Figure 6C). The results indicate that the presence of *Stvb-i* attenuates the damage caused by heat stress and that *Stvb-i* relates in regulation of heat-responsive pathway; this gene contributes to plant growth.

### Heat Response of *Stvb-i* and *Hsp70* Genes

Heat shock proteins 70 (Hsp70s) are important in the thermal response (Wang et al., 2004; Wahid et al., 2007), and are involved in viral infection and multiplication (Mayer, 2005; Nagy et al., 2011). We examined the expression of *Stvb-i* and four rice *Hsp70* genes in heated plants using quantitative RT-PCR. The transcripts of three *Hsp70*s—*Os03g60620*, *Os11g08470*, and *Os05g38530*—accumulate at 30 days after RSV inoculation (Jiang et al., 2014), and *Os05g38530* is reportedly upregulated by heat stress (42°C 10 h) (Hu et al., 2009). Another *Hsp70*, *Os11g47760*, encodes a protein highly homologous to Os03g60620, Os05g38530, and NbHsp70 (KX912913) of *N. benthamiana.* NbHsp70 is associated with multiplication of Chinese wheat mosaic furovirus in *N. benthamiana* and affects leaf development (Yang et al., 2017).

**FIGURE 7:**
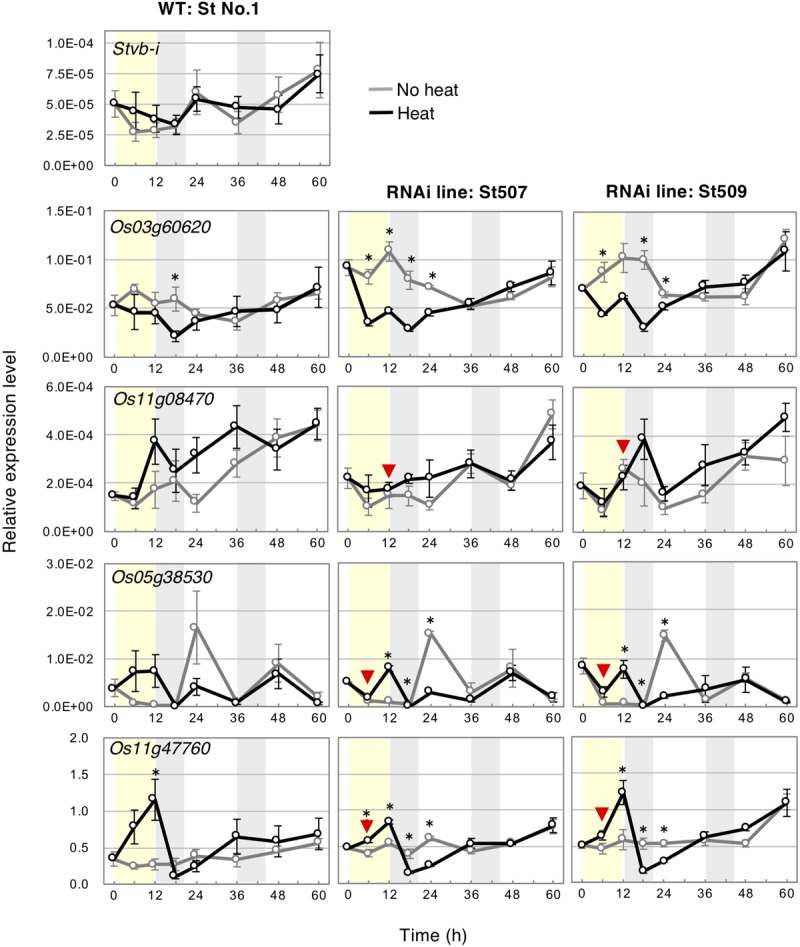
Effect of heat treatment on the expression of *Stvb-i* and *Hsp* genes in seedlings of WT St No. 1 and *Stvb-i*–silenced lines at 1.5-leaf stage. Relative expression levels were calculated from quantitative RT-PCR results using *OsGAPDH* as a reference and are shown as means ± SEM (*n* = 3). Red arrowheads indicate the unaffected gene expression in RNAi lines in comparison with WT in heat-treated plants. Asterisks show significant differences between heated and non-heated samples (Tukey–Kramer test, *p* < 0.05, *n* = 3). Light yellow shading represents 12-h heat treatment at 38°C. Light gray shading represents the 8-h dark period.

In the WT St No. 1, the *Stvb-i* expression level seemed to increase slightly in heated than in unheated plants and was about twice the level of unheated plants at 6 h; the expression levels became similar at later time points regardless of heat treatment (Figure 7). Silencing of *Stvb-i* significantly increased the expression level of *Os03g60620* at several time points and tended to slightly increase the expression level of *Os11g47760* at all time points (Supplementary Figure S8).

In *Stvb-i* silenced lines, unlike in WT, heat greatly decreased the expression of *Os03g60620* at 6–24 h (Figure 7). On the other hand, heat had no effect on the expression of *Os05g38530* and *Os11g047760* at 6 h, and on *Os11g08470* expression at 12 h (Figure 7). The results suggest that *Stvb-i* involves in heat-responsive control of the *Hsp70* genes, especially *Os03g60620*.

## Discussion

In response to various external stimuli and environmental stresses, plants modulate various signaling pathways in meristems that support plant development. In this study, we found that the RSV resistance gene *Stvb-i* in rice contributes to developmental homeostasis in meristems and focused on the role of this gene in resistance to heat and RSV.

We found that the function of *Stvb-i* paralogs is related to developmental homeostasis. The genes of this family are mainly expressed in immature tissues containing meristems, and their expression patterns differed at different growth stages. *Stvb-i* was specifically expressed in immature tissues of seedlings, tillers, and young panicles (Figure 4B). Its suppression inhibited plant growth for a long period from the vegetative to reproductive stages (Figures 5A,B and Supplementary Figure S5). The relationship between expression pattern and plant development was also observed for the *Stvb-i*–paralogous gene *Os11g31500* (Figures 4B, 5C and Supplementary Figure S7). These results suggest that the *Stvb-i* family genes affect plant growth and development. In particular, *Stvb-i* may support the growth of meristems. *Stvb-i* was highly expressed in calluses (Supplementary Figure S4), which are composed of cells capable of growth and division, similar to meristems.

In the seedling base of the resistant rice, the concentration and the spread of RSV were suppressed (Figure 1A). The mechanism of RSV resistance conferred by *Stvb-i* may be similar to that employed to produce virus-free plants from an infected plant by meristem-tip culture (Spangenberg et al., 1998; Razdan, 2003). This technique is based on virus elimination from newly generated cells in meristems (Spangenberg et al., 1998), possibly because cell growth rate is higher than viral multiplication rate owing to vigorous metabolism of the host cells (Razdan, 2003). *Stvb-i* may help to maintain cell growth rate even in the meristems infected with RSV, reducing RSV concentration at every cell division. This would result in rice plant growth quickly escaping from spreading RSV.

*Stvb-i* greatly affects the heat tolerance of plant growth. When exposed to high temperature, rice plants show various abnormal phenotypes: thin and short crown roots, chlorosis, wilting and stunting in seedlings, decrease in tiller number during vegetative growth, and defective panicle formation in the reproductive period (Krishnan et al., 2011). Similar abnormalities were observed at normal temperature in the *Stvb-i*–silenced plants (Figures 5A,B and Supplementary Figure S5A) and were exacerbated in plants exposed to high temperature for many days (Figure 6C and Supplementary Figure S5B). The role of *Stvb-i* in meristems is closely related to the response to heat stress.

Temperature above the optimum for growth impairs cell homeostasis (Bokszczanin et al., 2013), and plants regulate gene expression in response to heat (Liu et al., 2015). Heat shock proteins (Hsps) greatly contribute to cell homeostasis as molecular chaperones (Wang et al., 2004; Wahid et al., 2007). Hsp90s, a major Hsp family, regulate cell-cycle control, signal-transduction networks, and protein folding, trafficking, and degradation (Wang et al., 2004; Sangster and Queitsch, 2005). *Stvb-i* is similar to Hsp90 in the structure of its N-terminal domain, expression (constitutive under usual conditions), and function (contribution to recovery from heat stress and influence on plant development). *Stvb-i* may regulate heat-sensitive signaling cascades closely linked to meristem growth.

RNA virus requires host Hsp70s (Mayer, 2005; Nagy et al., 2011). Hsp70s function together with Hsp90s as molecular chaperones, particularly in RNA metabolism (Walters and Parker, 2015; Jacob et al., 2017). In RSV infection, the involvement of rice *Hsp70* genes, *Os03g60620*, *Os11g08470*, and *Os05g38530*, has been suggested (Jiang et al., 2014). *Stvb-i* silencing affected the expression of the *Hsp70s* (Figure 7 and Supplementary Figure S8) and growth of plant (Figures 5A,B), the results suggest a possibility that the four Hsp70s examined in this study function in plant development. The NbHsp70 (KX912913), homologous to Os03g60620, Os05g38530, and Os11g47760, is required for the normal growth and development of *N. benthamiana* (Yang et al., 2017). RSV may recruit Hsp70s for its multiplication and interfere with the native roles of Hsp70s, affecting rice meristem growth and causing systemic symptoms.

The RSV symptoms are similar to morphological changes in heat-damaged plants (Washio et al., 1967; Ling, 1972; Krishnan et al., 2011; Supplementary Figure S9). Growth stunting caused by RSV infection was significantly exacerbated by *Stvb-i*-silencing (Figure 5A). The results suggest that RSV multiplication may induce heat-like stress in meristematic cells and interfere with a cascade in which *Stvb-i* is involved. Probably, the mechanism of meristem growth conferred by *Stvb-i* functions to alleviate not only stress caused by high temperature, but also stress caused by RSV.

The major type of plant disease resistance genes encodes NBS-LRR proteins, which confer resistance to pathogens by eliciting hypersensitive response (programmed cell death) and thus preventing pathogen invasion and propagation, which results in selective pressure. The interaction between an NBS-LRR protein and pathogenic factor requires Hsp90 and is therefore temperature sensitive (Samuel, 1931; Kobayashi et al., 2014; Park and Seo, 2015; Qian et al., 2018). RSV resistance conferred by *Stvb-i* has remained stable over the past 50 years despite an increase in the number of days when the temperature exceeded the optimal temperature for rice seedling growth, providing ideal conditions for RSV multiplication (Jiang et al., 2014; weather data are from the Japan Meteorological agency^16^). A stable expression of *Stvb-i* (Figures 3B, 7), which contributes to such stress recovery of meristematic cells, will strongly and quickly suppress viral multiplication even under high pressures of RSV infection. Unlike in the case of the major resistance genes encoding NBS-LRR proteins, the viral resistance conferred by *Stvb-i* is attributable to the basic response to heat stress in host meristematic cells. Although *Stvb-i* is unable to completely suppress RSV multiplication, it certainly confers durable and sustainable RSV tolerance combined with heat insensitivity.

Our study has demonstrated that rice acquires sustainable dual tolerance to abiotic and biotic stresses through the stability of plant growth. In the context of global warming, exploration of genes involved in the metabolic and signaling systems that maintain meristem growth will be conducive to conferring multi-tolerance in plants. Improvement of developmental stability will help secure the productivity of crops under diverse environmental conditions.

## Data Availability Statement

The datasets generated for this study are included in the article/Supplementary Materials.

## Author Contributions

YH-S and KH performed the experiments and analyzed the data. YH-S designed the experiments and wrote the manuscript.

## Conflict of Interest

The authors declare that the research was conducted in the absence of any commercial or financial relationships that could be construed as a potential conflict of interest.

## Footnotes

1 http://www.knowledgebank.irri.org/

2 http://www.naro.affrc.go.jp/org/niaes/aws/

3 http://www.naro.affrc.go.jp/harc/kisyo/index.html

4 https://vectorlabs.com

5 https://www.keyence.com

6 https://www.thermofisher.com/jp/ja/home.html

7 http://www.ebi.ac.uk/interpro/

8 http://rice.plantbiology.msu.edu/

9 https://www.genetyx.co.jp

10 https://www.qiagen.com/us/

11 https://www.thermofisher.com/jp/ja/home.html

12 https://www.takara-bio.com

13 https://www.n-genetics.com

14 https://www.toyobo-global.com

15 https://www.ssri.com

16 http://www.data.jma.go.jp/
