# Supplementary figures and images for: Stvb-i, a Rice Gene Conferring Durable Resistance to Rice stripe virus, Protects Plant Growth From Heat Stress
Source: Front Plant Sci. 2020 May 8;11:519. doi: 10.3389/fpls.2020.00519 (PMC7225774; doi:10.3389/fpls.2020.00519)

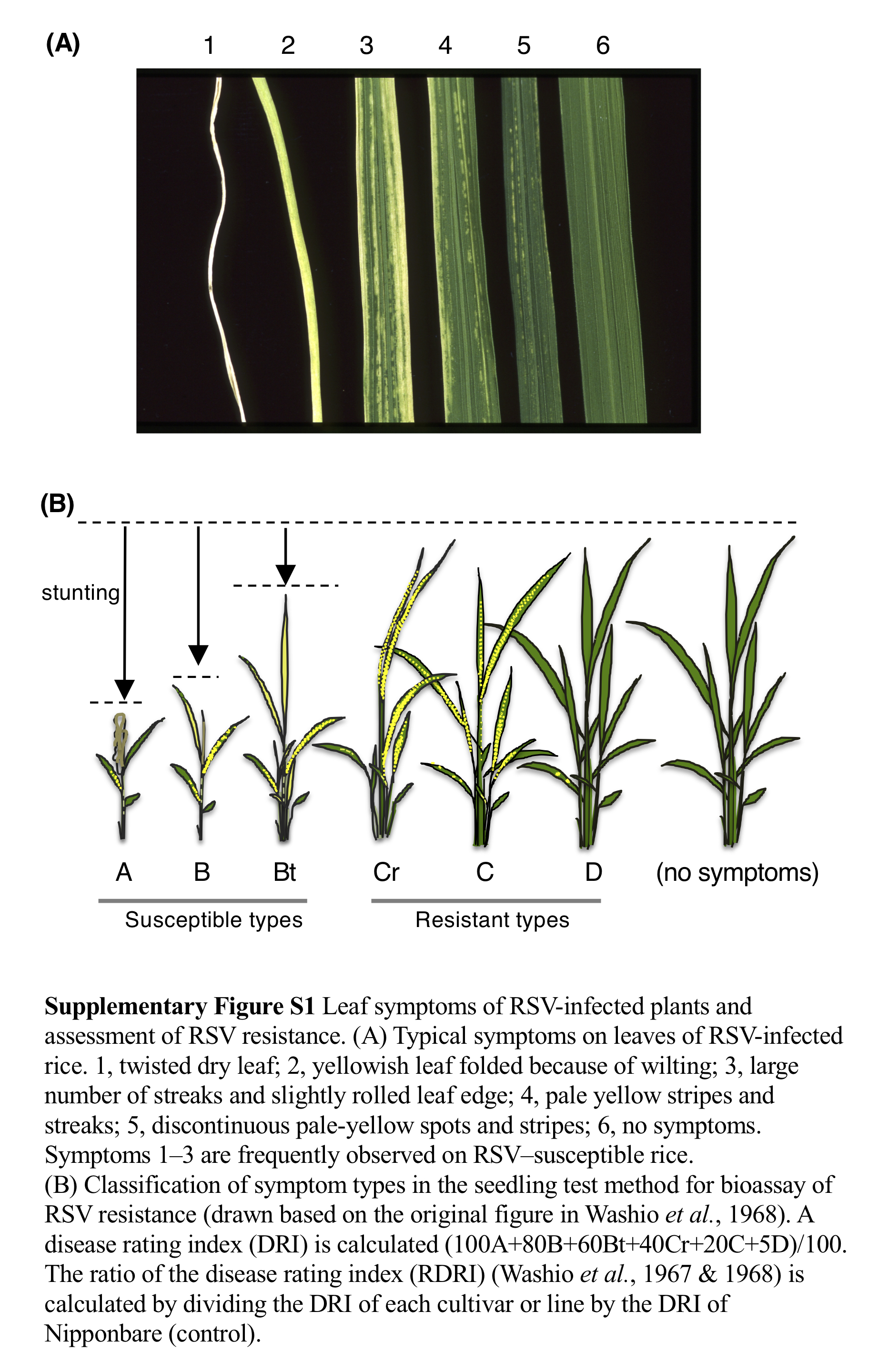

Supplement: Supplementary file 1 [file Image_1.jpg]

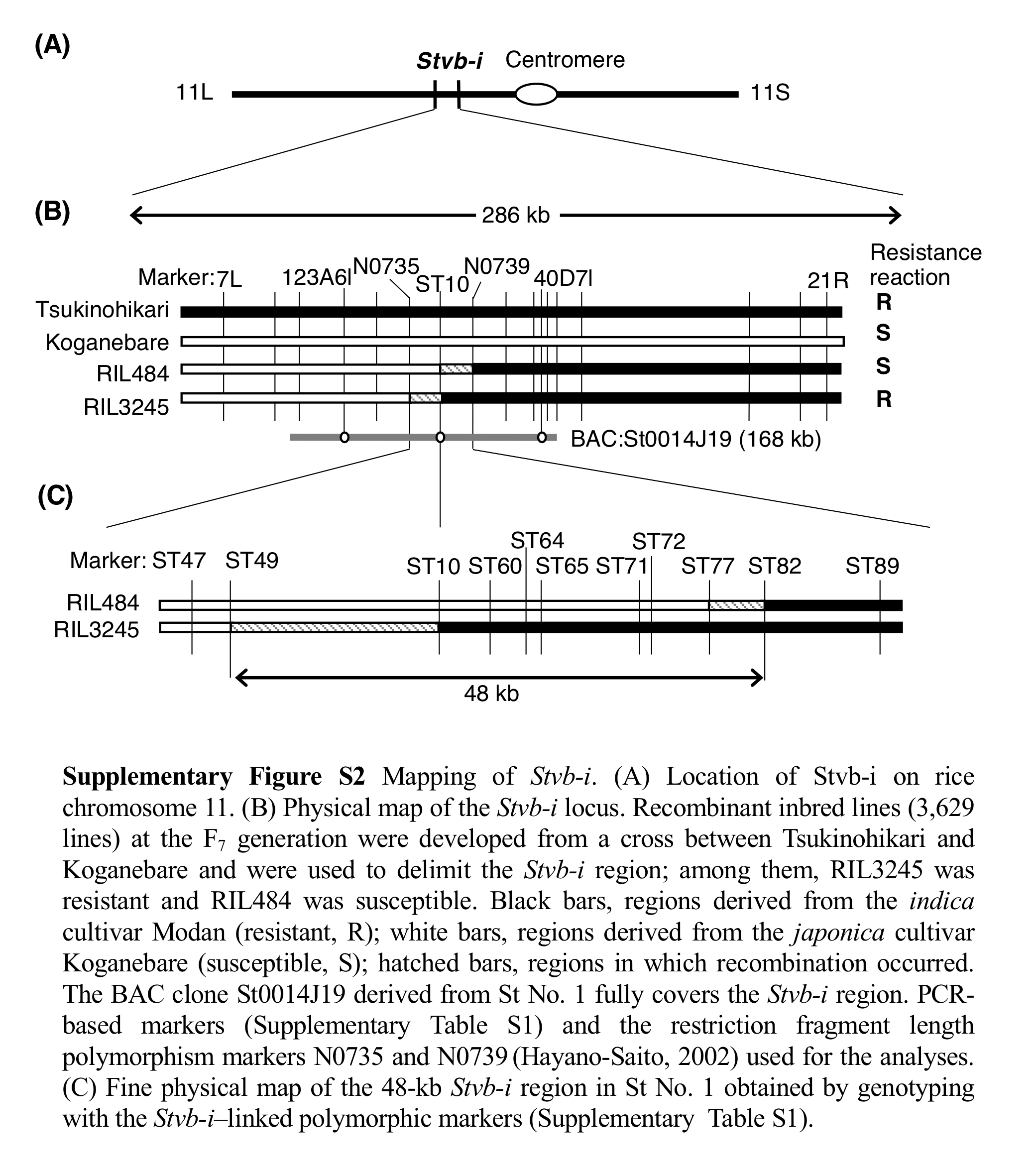

Supplement: Supplementary file 2 [file Image_2.jpg]

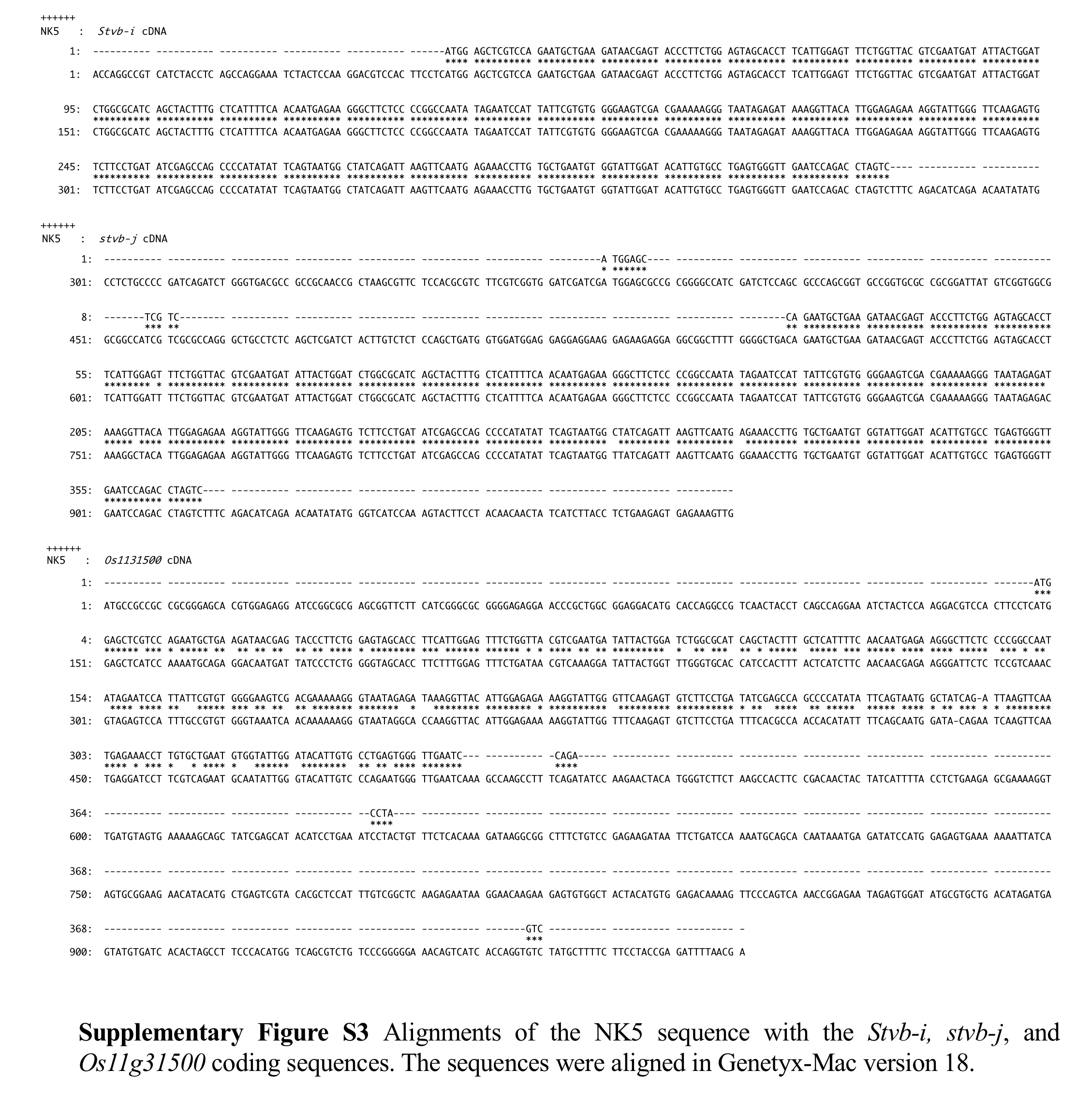

Supplement: Supplementary file 3 [file Image_3.JPEG]

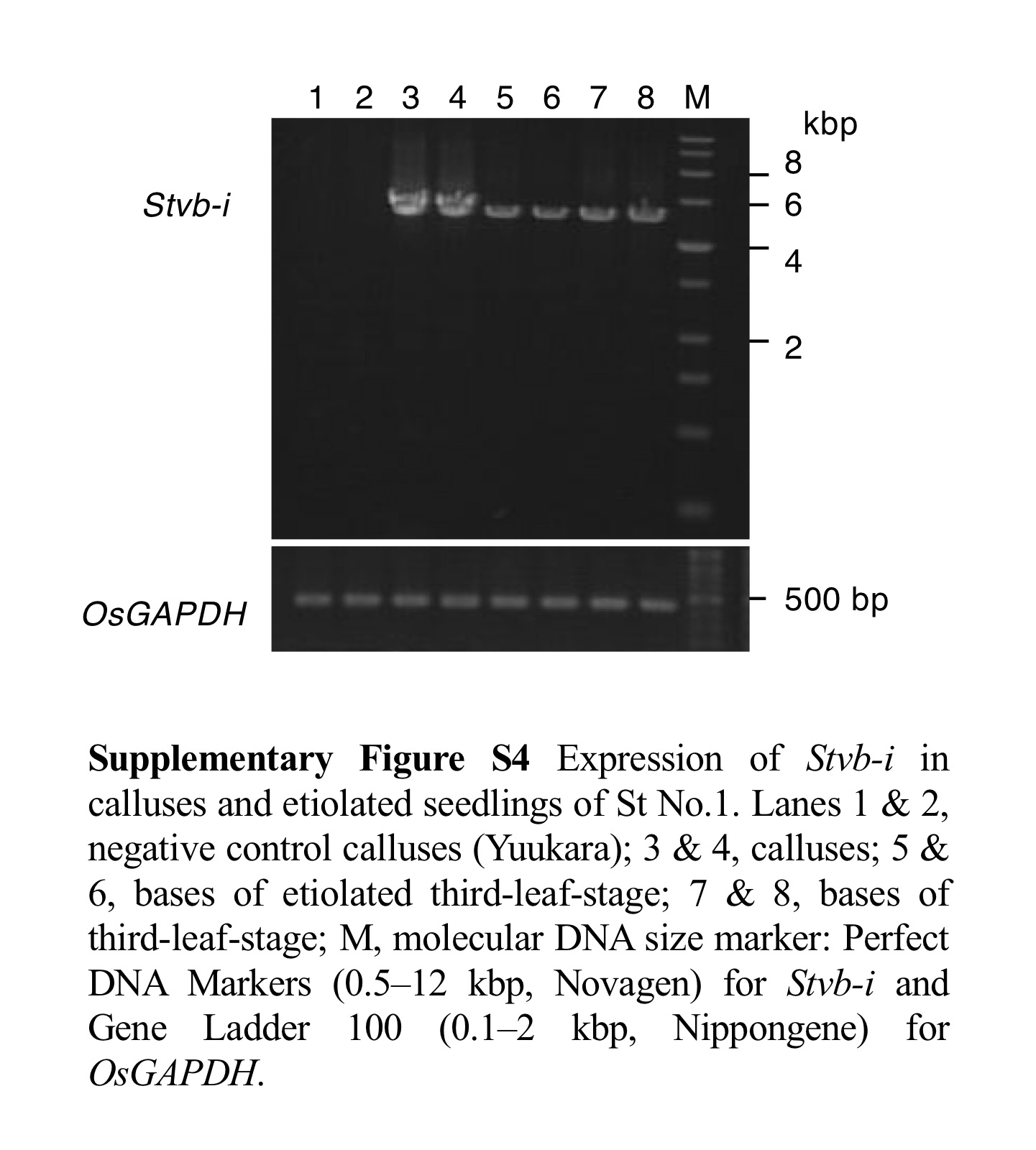

Supplement: Supplementary file 4 [file Image_4.JPEG]

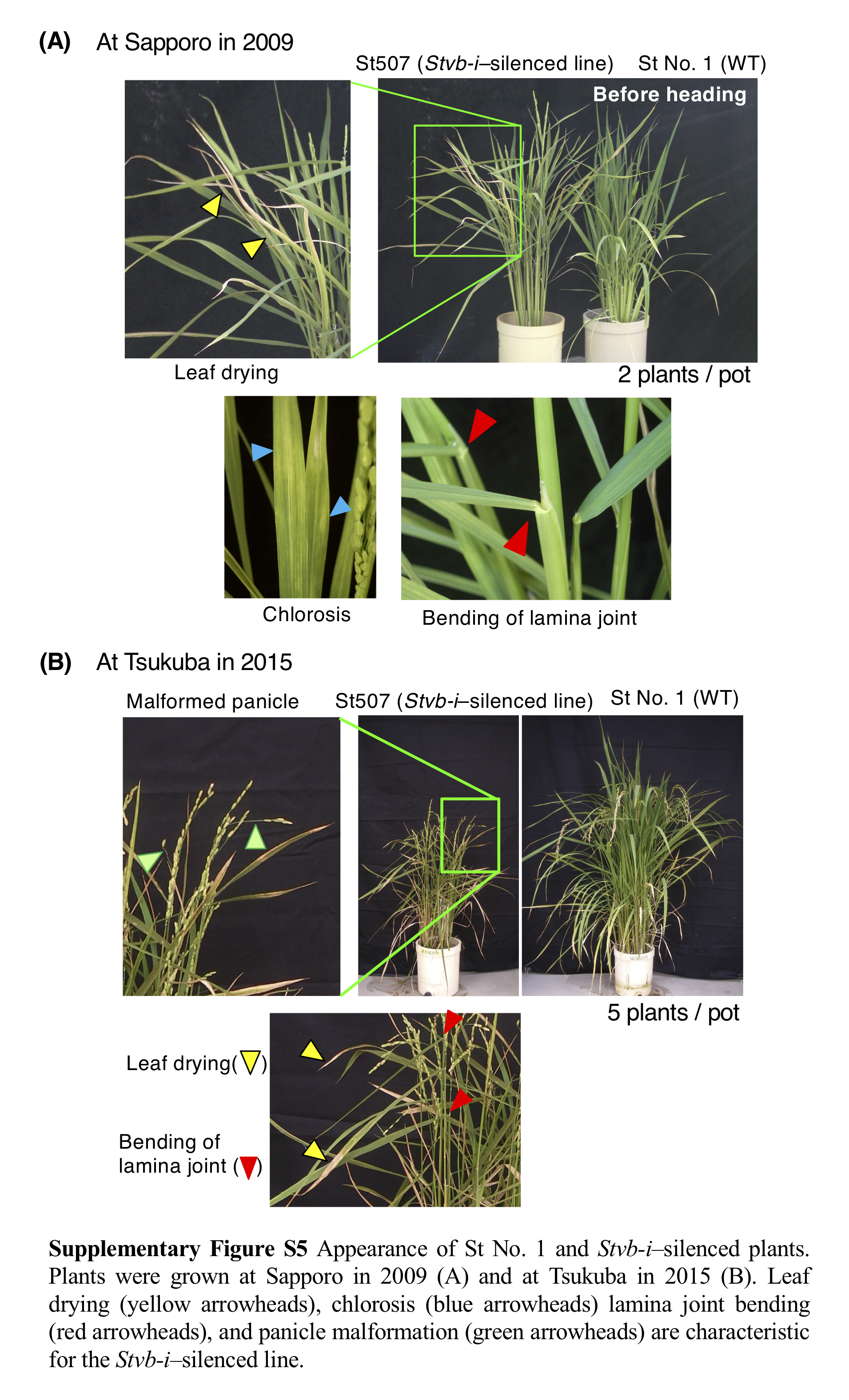

Supplement: Supplementary file 5 [file Image_5.JPEG]

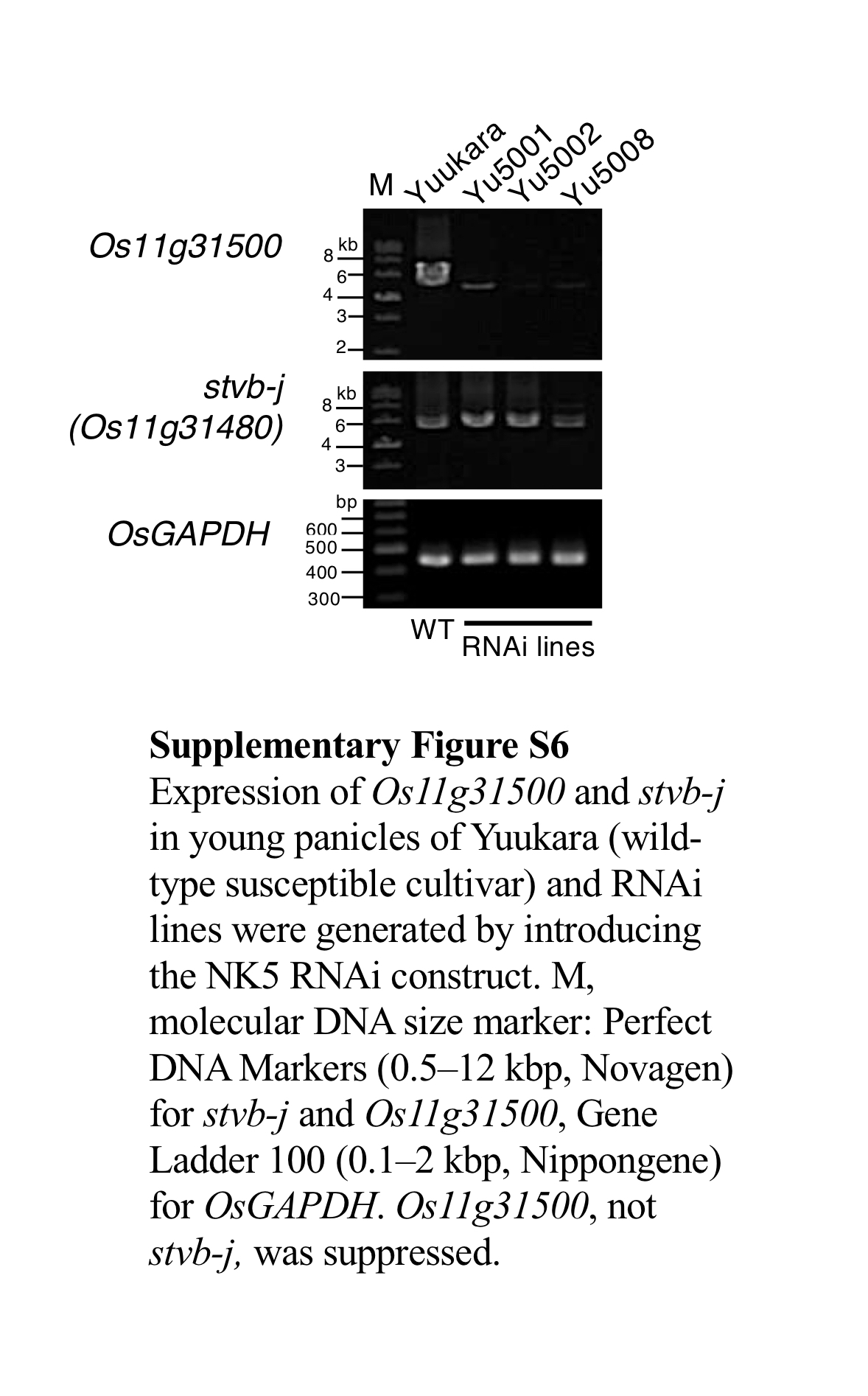

Supplement: Supplementary file 6 [file Image_6.JPEG]

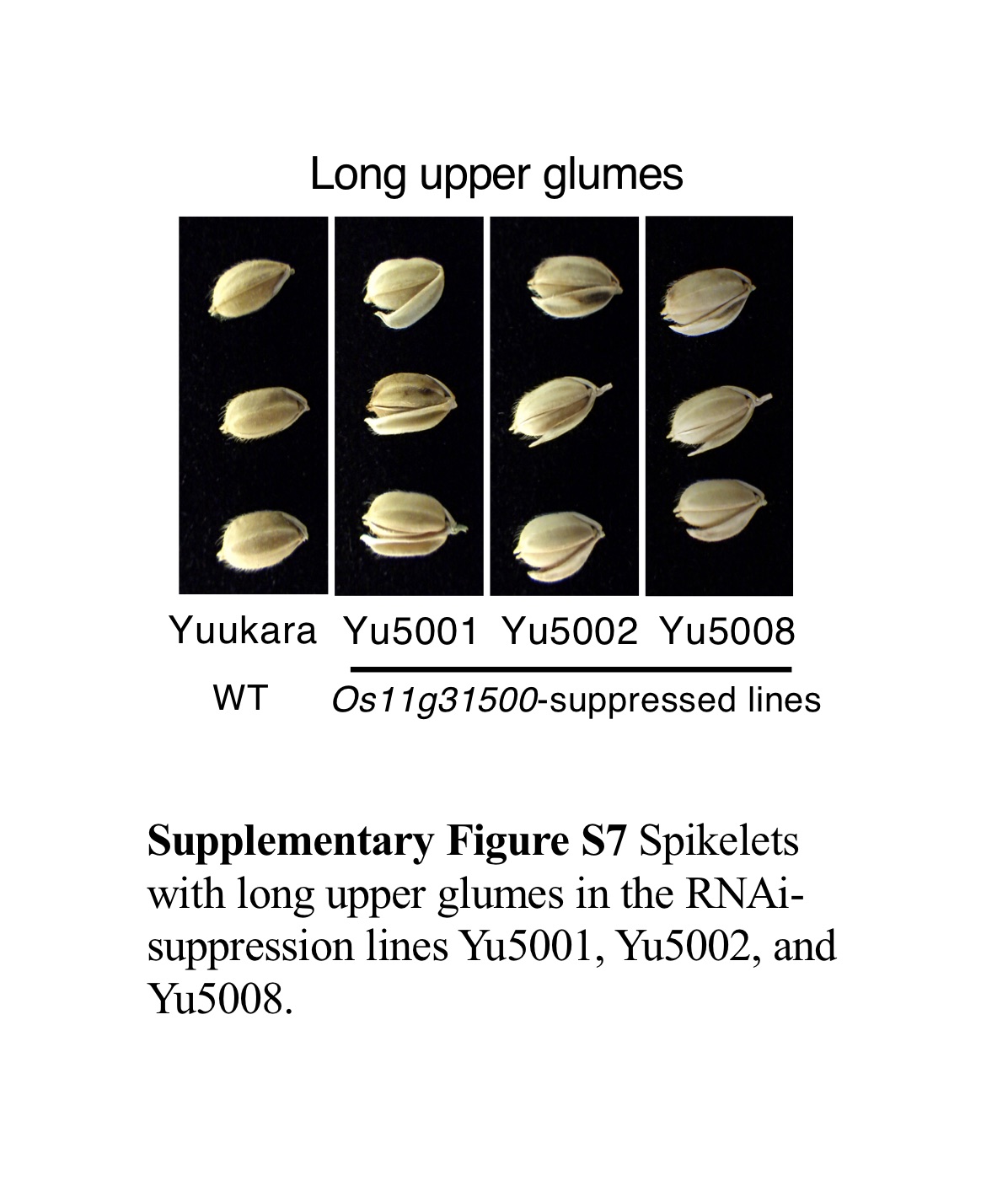

Supplement: Supplementary file 7 [file Image_7.JPEG]

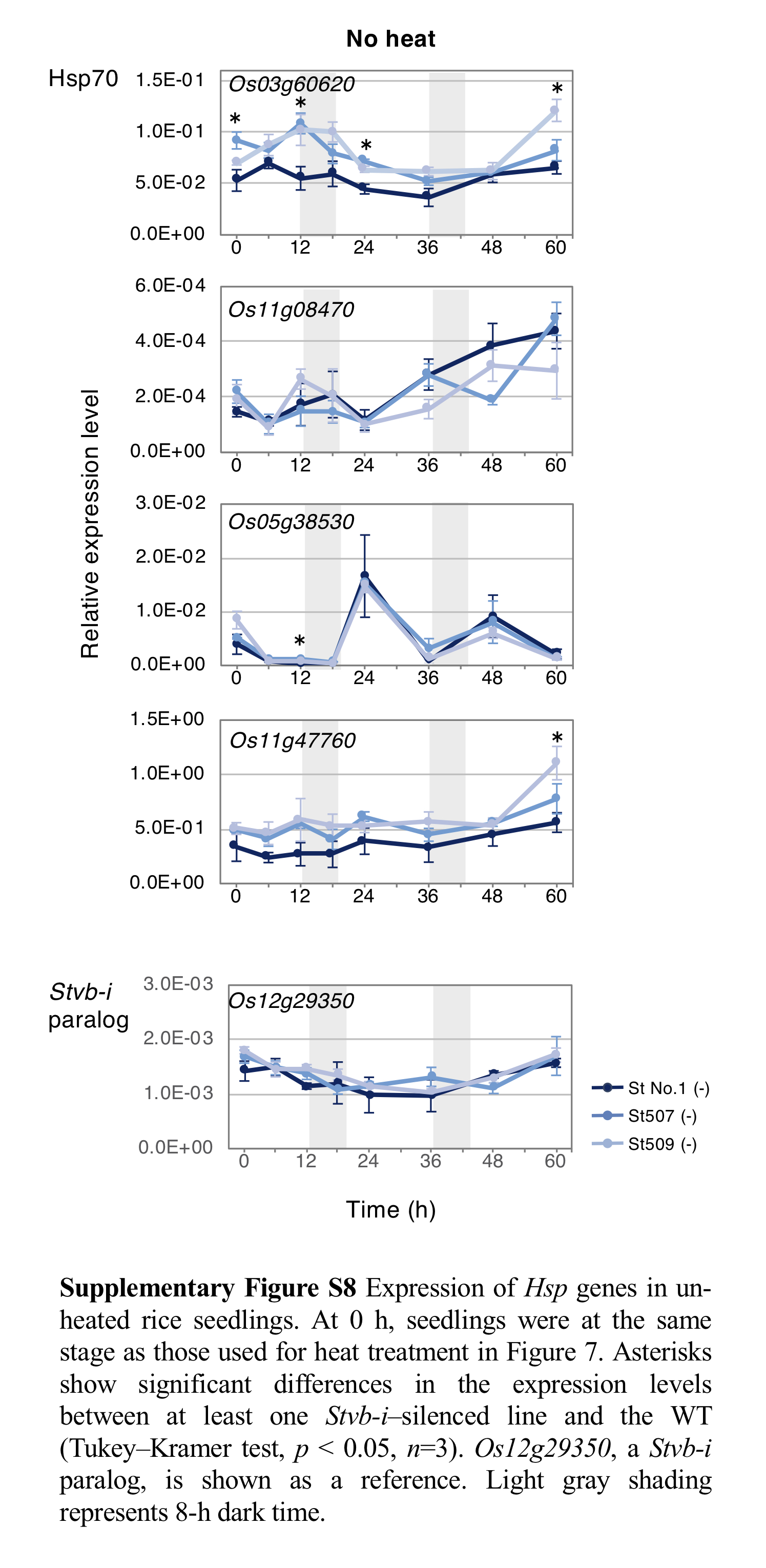

Supplement: Supplementary file 8 [file Image_8.JPEG]

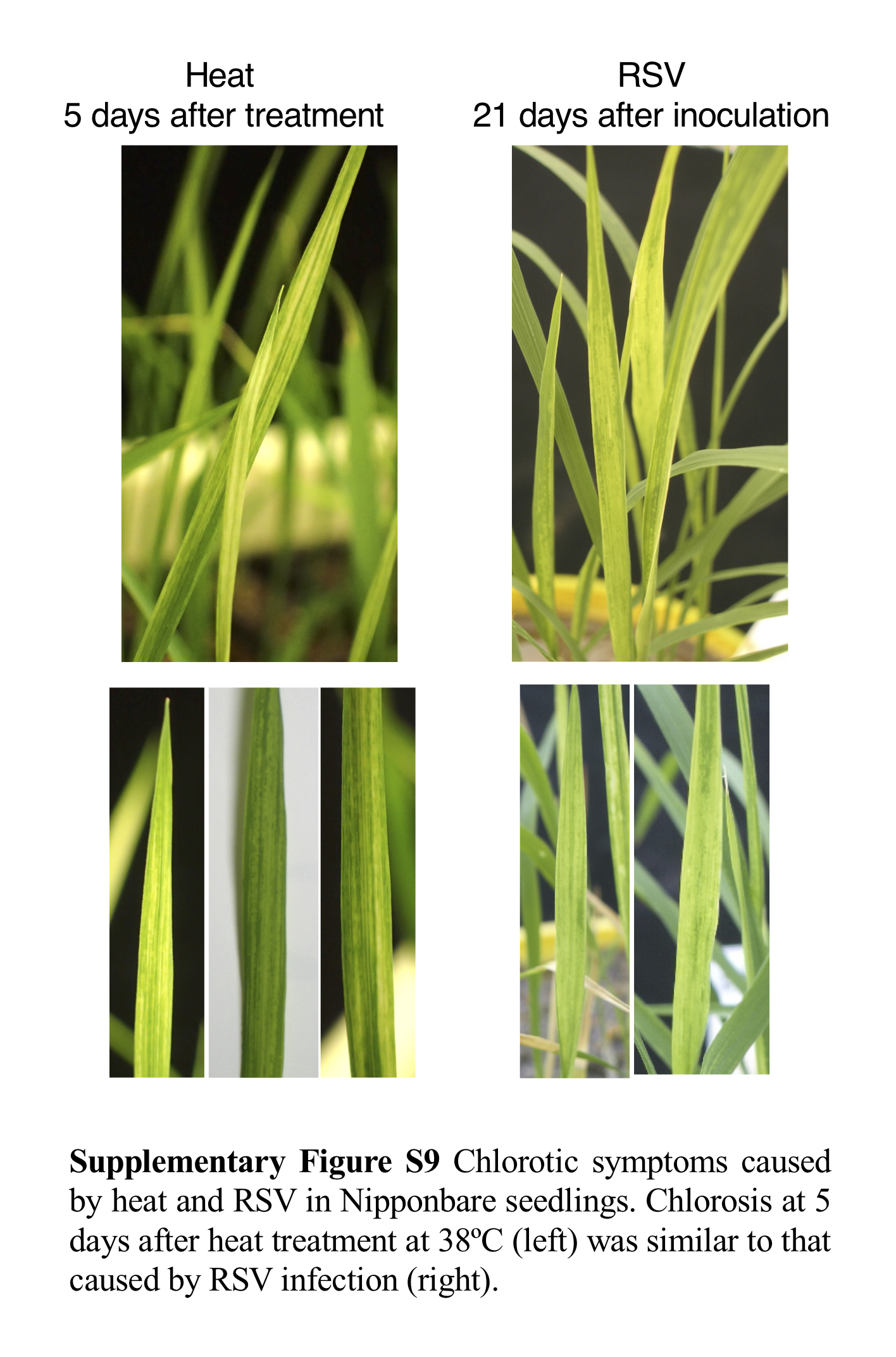

Supplement: Supplementary file 9 [file Image_9.JPEG]
